# Effect of Robot-Assisted Neuroendoscopic Hematoma Evacuation Combined Intracranial Pressure Monitoring for the Treatment of Hypertensive Intracerebral Hemorrhage

**DOI:** 10.3389/fneur.2021.722924

**Published:** 2021-12-02

**Authors:** Shiqiang Wu, Heping Wang, Junwen Wang, Feng Hu, Wei Jiang, Ting Lei, Kai Shu

**Affiliations:** Department of Neurosurgery, Tongji Hospital, Tongji Medical College, Huazhong University of Science and Technology, Wuhan, China

**Keywords:** hypertensive intracerebral hemorrhage, neuroendoscopic hematoma evacuation, intracranial pressure monitoring, Remebot robot, clinical effect

## Abstract

**Objective:** This study aimed to investigate the clinical efficacy of robot-assisted neuroendoscopic hematoma evacuation combined intracranial pressure (ICP) monitoring for the treatment of hypertensive intracerebral hemorrhage (HICH).

**Patients and Methods:** A retrospective analysis of 53 patients with HICH undergoing neuroendoscopic hematoma evacuation in our department from January 2016 to December 2020 was performed. We divided the patients into two groups: the neuroendoscopic group (*n* = 32) and the robot-assisted neuroendoscopic combined ICP monitoring group (*n* = 21). Data on clinical characteristics, treatment effects, and outcomes were retrospectively reviewed and analyzed between these two groups.

**Results:** The operation time of the procedure of the neuroendoscopic group was significantly longer than that of the robot-assisted neuroendoscopic combined ICP-monitoring group (mean time 153.8 ± 16.8 vs. 132.8 ± 15.7 min, *P* < 0.001). The intraoperative blood loss was significantly less in the robot-assisted neuroendoscopic combined ICP-monitoring group than in the neuroendoscopic group (215.4 ± 28.3 vs. 190.1 ± 25.6 ml, *P* = 0.001). However, the patients undergoing neuroendoscopic had a comparable hematoma clearance rate with those undergoing robot-assisted neuroendoscopic combined ICP monitoring (85.2 ± 4.8 vs. 89.2 ± 5.4%, *P* = 0.997). The complications rate was greater in the endoscopic group (25%) than in the robot-assisted neuroendoscopic combined ICP-monitoring group (9.5%) but without significant difference (*P* = 0.159). We also found that the dose of used mannitol was significantly less in the ICP monitoring group (615.2 ± 63.8 vs. 547.8 ± 65.3 ml, *P* < 0.001) and there was a significant difference in modified Rankin scale (mRS) score at discharge, patients with less mRS score in the robot-assisted neuroendoscopic combined ICP monitoring group than in the neuroendoscopic group (3.0 ± 1.0 vs. 3.8 ± 0.8, *p* = 0.011). Patients undergoing robot-assisted neuroendoscopic combined ICP monitoring had better 6-month functional outcomes, and there was a significant difference between the two groups (*p* = 0.004). Besides, multivariable analysis shows younger age, no complication, and robot-assisted neuroendoscopic combined ICP monitoring were predictors of 6-month favorable outcomes for the patients with HICH.

**Conclusion:** Robot-assisted neuroendoscopic hematoma evacuation combined with ICP monitoring appears to be safer and more effective as compared to the neuroendoscopic hematoma evacuation in the treatment of HICH. Robot-assisted neuroendoscopic hematoma evacuation combined with ICP monitoring might improve the clinical effect and treatment outcomes of the patients with HICH.

## Introduction

Hypertensive intracerebral hemorrhage (HICH) is a common serious cerebrovascular disease with high mortality and morbidity rate, which frequently occurs in the middle-aged and elderly population, with a peak incidence in winter and spring (1–3). Due to the influence of long-term hypertension, it can cause arteriosclerosis in the brain, decrease the elasticity of the blood vessel wall, increase the brittleness, cause cellulose necrosis, and promote the formation of a miliary aneurysm. Once the blood pressure rises, the aneurysm will rupture and cause cerebral hemorrhage. Owing to the harmfulness and the risks of HICH, the patients should get timely and effective treatment (4, 5).

At present, surgical treatment and conservative medical methods are the two main options for patients with HICH, but there is no uniform standardized treatment method. The surgical methods mainly include conventional craniotomy, small-bone window craniotomy, stereotactic aspiration, neuroendoscopic hematoma evacuation, and so on (6, 7). The principle of operation is to clear the hematoma and promote the recovery of neurological function. With the recent advances in endoscopic technique, neuroendoscopes have been used for the treatment of intracranial cysts and hydrocephalus and as an adjunct during microsurgical tumor removal. Meanwhile, neuroendoscopic hematoma evacuation has been applied widely in the treatment of patients with HICH, which is less invasive than craniotomy and can also easily achieve hemostasis of the bleeding vessels (8, 9). However, neuroendoscopic hematoma evacuation also has its inherent shortcomings such as a narrow surgical window results in a low-removal rate, sometimes cannot reach the designated position accurately and rapidly, and should be performed by a skilled surgeon. To the best of our knowledge, there have been several reports on the use of robotic devices in neuroendoscopic surgery. Zimmermann et al. (10) demonstrated their preliminary experience with robot-assisted navigated endoscopic third ventriculostomies, the authors proved for the first time that robot assistance allows highly exact and repeatable positioning of a rigid endoscope with an accuracy of 50 mm, a very smooth and slow insertion of the endoscope within the brain tissue. According to this, neuro-navigation and robotic technologies have been developed and used in surgical procedures to reduce brain injury and improve clinical outcomes and prognosis of patients with HICH (11).

In the meantime, patients still have many complications after the operation, including rehemorrhage, brain swelling, and cerebral ischemia that can cause increased intracranial pressure (ICP), thus, ICP monitoring in the treatment of patients with HICH is of great importance for preventing the occurrence of complications. Many scholars have reported that ICP monitoring was associated with a good prognosis (12, 13). In our study, we retrospectively reviewed 53 patients with HICH undergoing neuroendoscopic hematoma evacuation in the Tongji Hospital, the purpose of this study was to investigate the clinical efficacy of robot-assisted neuroendoscopic hematoma evacuation and ICP monitoring for the treatment of HICH.

## Materials and Methods

### Patient Population

This retrospective study was permitted and sponsored by the Tongji Hospital, Tongji Medical College, Huazhong University of Science and Technology. January 2016 to December 2020, we reviewed 53 patients who underwent neuroendoscopic hematoma evacuation at the Department of Neurosurgery, Tongji Hospital. The patients were divided into two groups according to the surgical strategy: the neuroendoscopic group (*n* = 32) and the robot-assisted neuroendoscopic combined ICP monitoring group (*n* = 21). The clinical data regarding patient age, sex, neuroimaging features, outcomes, postoperative complications, and the duration of the operation procedure and hospitalization were retrospectively analyzed. The inclusion criteria were as follows: patients with HICH were confirmed by CT scan with hematoma volume > 30 ml; the past medical history of hypertension; disease onset within 24 h and GCS score ≥ 4. The exclusion criteria were as follows: hemorrhage caused by aneurysm, trauma, tumor, arteriovenous malformation, venous sinus thrombosis; preoperative administration of antiplatelet or anticoagulant drugs; patients with severe systematic comorbidities.

### Surgical Techniques

All the surgeries were performed by the same neurosurgeon, Professor Kai SHU, Department of Neurosurgery, Tongji Hospital, Tongji Medical College, Huazhong University of Science and Technology, who performed 300 operations of robotic surgery every year. Thirty-two patients underwent neuroendoscopic hematoma evacuation, and 21 patients underwent robot-assisted neuroendoscopic hematoma evacuation combined with ICP monitoring. All the patients underwent CT angiography to exclude arteriovenous malformation and aneurysm before surgery. We used the Glasgow Coma Scale (GCS) and the National Institutes of Health Stroke Scale (NIHSS) to assess the neurological function of patients with HICH at admission. The Coniglobus formula was used to estimate the hematoma volumes, that is, volume = (length × width × thickness)/2. And the hematoma clearance rate =100- (postoperative volume)/(preoperative volume) × 100. All the patients were followed up for 6 months.

For the neuroendoscopic hematoma evacuation procedures, after skin disinfection, a burr hole was made on the skull nearest to the hematoma for patients with lobar hemorrhage or the forehead at the site 1.5 cm inside the hairline and 2.5–3.5 cm from the midline for the patients with basal ganglia hemorrhage. A small-bone window was opened with a size of 3 cm diameter by using the milling cutter. Then, a sheath with stylet inside was inserted after the dura matter was incised. Based on the location and volume of the hematoma, the depth and orientation of the sheath were determined by the experience of the surgeon. The stylet was removed and an endoscope with 0 or 30 degrees was inserted once the sheath reached the hematoma. The hematoma was removed under neuroendoscope. The hematoma cavity was filled with absorbable hemostatic gauze, and a monopolar electrocautery probe was used for hemostasis when artery bleeding was seen. After the hematoma was evacuated, the drainage tube was inserted into the hematoma cavity and we sutured and disinfected the wound.

**Figure 1:**
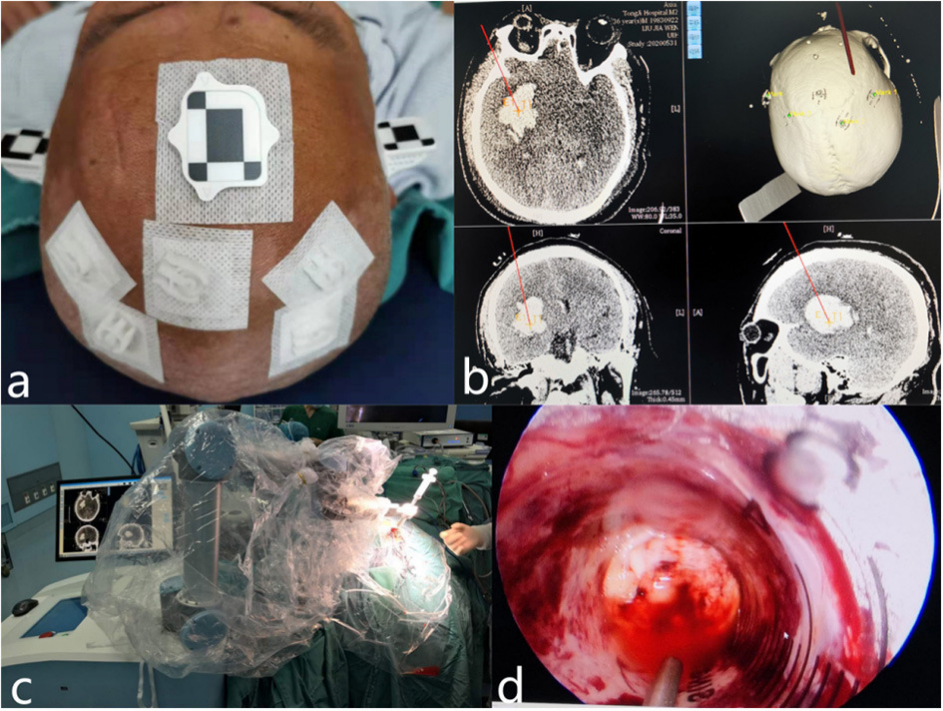
Representative images of robot-assisted neuroendoscopic hematoma evacuation combined ICP monitoring surgical workflow. **(a)** Markers were attached to the temple and forehead of the patient, and then CT scans were performed before the operation. **(b)** The entry point, the range of hematoma, and optimal trajectory were carefully planned by the surgeon. **(c)** After the dura matter was incised, a sheath with stylet inside was inserted into the hematoma cavity with the assist of a Remebot robot. **(d)** The hematoma was removed under neuroendoscope.

For the robot-assisted neuroendoscopic hematoma evacuation combined ICP monitoring procedures, markers were attached to the temple and forehead of the patient, and then CT scans were performed before operation. All images were copied to the Remebot robot system (developed by Beijing Baihuiweikang Technology Company, and approved by the National Medical Products Administrations, China), and then the entry point, the range of hematoma, and optimal trajectory were carefully planned by the surgeon. The head of the patient was immobilized in a Mayfield clamp after general anesthesia. After accurate registration, a burr hole was drilled based on preoperative planning. After the dura matter was incised, a sheath with stylet inside was inserted into the hematoma cavity with the assist of a Remebot robot. The methods of removing and aspirating hematoma, hemostasis, and suturing were the same as the treatment in the neuroendoscopic hematoma evacuation group. And we inserted a drainage tube with the Codman ICP monitoring probe to the ventricle or hematoma cavity (Figure 1). The drainage tube with the Codman ICP monitoring probe was inserted into the ventricle for patients who were complicated with ventricular hemorrhage. The drainage tube with the Codman ICP monitoring probe was inserted into the hematoma cavity for patients with HICH with no involvement of the ventricular system. Management of different dosages of mannitol was guided by ICP changes: for the patients with ICP higher than 25 mmHg, 125 ml of 20% mannitol every 6 h was used to decrease ICP to maintain ICP lower than 15 mmHg, also emergent CT scan would be performed if necessary; for ICP between 15 and 25 mmHg, 125 ml of 20% mannitol compound was intravenously injected every 8–12 h; and for ICP between 0 and 15 mmHg, it would be considered as normal.

### Statistical Analysis

Statistical analysis was performed using SPSS Statistics 22.0 (IBM Corporation, USA). Data are described as $\bar{\text{x}}$ ± *s*. The intergroup comparison was performed using the Student's *t*-test and the chi-squared test. Predictors of 6-month favorable outcomes were identified by using multiple-variable logistic regression (variables with statistical significance in univariate analysis were entered into a multivariate analysis). *p* < 0.05 was considered to be statistically significant.

## Results

Baseline characteristics are all shown in Table 1. A total of 53 patients were identified, with 34 men (64.2%) and 19 women (35.8%). The mean age of the population was 56.5 ± 9.1 years. There were no significant differences in patient age, sex, the GCS score, systolic pressure admission, the NIHSS score, localization of hematoma, hematoma volume, or midline shift between the neuroendoscopic group, and the robot-assisted neuroendoscopic hematoma evacuation combined ICP monitoring group.

**Table 1:** Summary of the baseline clinical characteristics of the patients.

**Parameters**	**Neuroendoscopic group (*n* = 32)**	**Robot-assisted neuroendoscopic combined ICP monitoring group (*n* = 21)**	****χ**^2^/*t*-value**	***P*-value**
Age (mean ± SD), years	56.2 ± 9.3	57.3 ± 8.9	0.195	0.577
Sex ratio (male/female)	21:11	13:8	0.076	0.782
GCS score	7.1 ± 2.1	7.8 ± 2.4	1.122	0.866
Systolic pressure admission (mmHg)	175.4 ± 17.3	178.9 ± 19.8	0.680	0.750
NIHSS score	11.2 ± 2.9	10.5 ± 2.7	−0.883	0.191
Left side	14	9	0.004	0.949
Hematoma volume (ml)	45.8 ± 15.4	46.9 ± 16.8	0.245	0.596
Location	0.707			0.699
Lobar	10	8		
Basal ganglia region	15	10		
Cerebellum	3	2		
Intraventricular extension	4	1		
Midline shift (mm)	8.1 ± 1.9	8.4 ± 1.8	0.574	0.716

The operation time of the procedure of the neuroendoscopic group was significantly longer than that of the robot-assisted neuroendoscopic combined ICP-monitoring group (mean time 153.8 ± 16.8 vs. 132.8 ± 15.7 min, *P* < 0.001). The intraoperative blood loss was significantly less in the robot-assisted neuroendoscopic combined ICP-monitoring group than in the neuroendoscopic group (215.4 ± 28.3 vs. 190.1 ± 25.6 ml, *P* = 0.001). However, the patients undergoing neuroendoscopic had a comparable hematoma clearance rate with those undergoing robot-assisted neuroendoscopic combined ICP monitoring (85.2 ± 4.8 vs. 89.2 ± 5.4%, *P* = 0.997). The complications rate was greater in the endoscopic group (25%) than in the robot-assisted neuroendoscopic combined ICP-monitoring group (9.5%) but without significant difference (*p* = 0.159). Complications occurred in eight cases of the neuroendoscopic group, including four patients with pulmonary infection, two with digestive tract hemorrhage, one with bleeding recurrence, and one who experienced a seizure. In the robot-assisted neuroendoscopic combined ICP monitoring group, one patient experienced pulmonary infection and one patient with digestive tract hemorrhage. The postoperative duration of hospitalization of the neuroendoscopic group was significantly longer than the robot-assisted neuroendoscopic combined ICP monitoring group (mean 13.8 ± 3.3 vs. 11.1 ± 2.8 days, *P* = 0.016).

**Figure 2:**
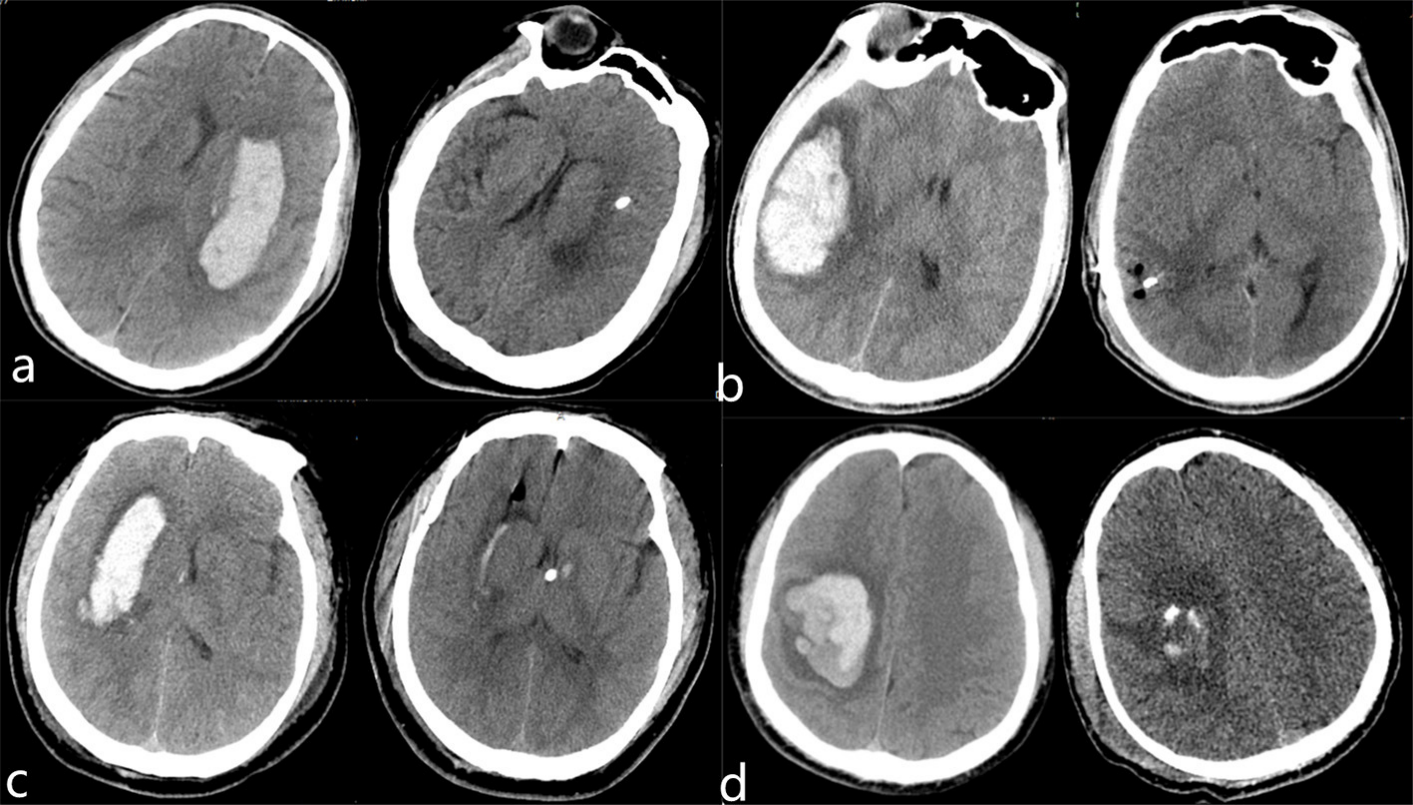
Preoperative and postoperative CT images in robot-assisted neuroendoscopic hematoma evacuation combined ICP monitoring of representative patients with HICH **(a–d)**. **(a)** A 62-year-old female was presented with a headache and right hemiplegia for 4 h. The hematoma was removed completely and the symptoms of headache were improved obviously. Continuous rehabilitation training was given to the patient after discharge. **(b)** A 54-year-old male presented with a headache for 10 h. Preoperative CT shows right temporal lobe hemorrhage that has been evacuated totally. **(c)** A 65-year-old male was presented with consciousness disturbance for 5 h. Preoperative CT scan shows right basal ganglia hemorrhage, which has been evacuated and we inserted a drainage tube with the Codman ICP monitoring probe to the ventricle. **(d)** A 73-year-old male was presented with left hemiplegia for 3 h. Preoperative CT shows right parietal lobe hemorrhage, which has been evacuated and we inserted a drainage tube with the Codman ICP monitoring probe to the hematoma cavity. Continuous rehabilitation training was given to the patient after they were discharged.

We also used the modified Rankin scale (mRs) to evaluate the neurological function recovery state of the patients. We found that the dose of used mannitol was significantly less in the ICP monitoring group (615.2 ± 63.8 vs. 547.8 ± 65.3 ml, *P* < 0.001), and there was a significant difference in mRS score at discharge, patients with less mRS score in the robot-assisted neuroendoscopic combined ICP monitoring group than in the neuroendoscopic group (3.0 ± 1.0 vs. 3.8 ± 0.8, *p* = 0.011). Moreover, there was no significant difference in mortality rate between the two groups. The patients were followed up for 6 months, and we utilized Glasgow Outcome Scale to assess the clinical outcome of the patients. In the endoscopic group, 16 patients had a good recovery, 13 patients had moderate disability, one patient was with a vegetative state, and two patients died. In the robot-assisted neuroendoscopic combined ICP-monitoring group, 15 patients had a good recovery, four patients had moderate disability, one patient was with a vegetative state, and one patient died. Patients undergoing robot-assisted neuroendoscopic combined ICP monitoring had better 6-month functional outcomes, and there was a significant difference between the groups (*P* = 0.004). These results are all summarized in Table 2. Table 3 summarizes the results from single and multiple logistic regression of Predictors of 6-month favorable outcomes. On single-variable logistic regression, younger age, less hematoma volume, not deep location, no complication, and robot-assisted neuroendoscopic hematoma combined with ICP monitoring were independent predictors of 6-month favorable outcomes. Sex, GCS score, systolic pressure admission, NIHSS score, the bleeding side of brain hemisphere, midline shift, operation time, intraoperative blood loss, hematoma clearance rate, a dose of mannitol, and postoperative duration of hospitalization were not significant prognostic factors for survival (*P* > 0.05). Multivariable analysis identified younger age, no complication and robot-assisted neuroendoscopic hematoma combined with ICP monitoring were predictors of 6-month favorable outcomes. There are four typical examples of the robot-assisted neuroendoscopic combined ICP-monitoring group in Figure 2.

**Table 2:** Comparison of postoperative data and clinical outcomes in the two groups of patients.

**Parameters**	**Neuroendoscopic group (*n* = 32)**	**Robot-assisted neuroendoscopic combined ICP monitoring group (*n* = 21)**	****χ**^2^/*t*-value**	***P-*value**
Operation time (min)	153.8 ± 16.8	132.8 ± 15.7	−4.566	<0.001^*^
Intraoperative blood loss (ml)	215.4 ± 28.3	190.1 ± 25.6	−3.303	0.001^*^
Hematoma clearance rate, %	85.2 ± 4.8%	89.2 ± 5.4%	2.824	0.997
Dose of mannitol (ml)	615.2 ± 63.8	547.8 ± 65.3	−3.727	<0.001^*^
Complications rate	8/32	2/21	1.984	0.159
Postoperative duration of hospitalization, days	13.8 ± 3.3	11.1 ± 2.8	−3.088	0.016^*^
mRS score at discharge	3.8 ± 0.8	3.0 ± 1.0	−3.223	0.011^*^
Mortality rate	2	1	0.053	0.819
Glasgow outcome scale after 6 months	3.86 ± 1.18	4.48 ± 1.16	1.927	0.033^*^

* *Statistically significant*.

**Table 3:** Predictors of 6-month favorable outcomes.

**Independent variable**	**Single variable logistic regression**			**Multiple logistic regression**		
	***p*-value**	**OR**	**95% CI**	***p*-value**	**OR**	**95% CI**
Age	0.001^*^	0.92	[0.86, 0.99]	0.013^*^	0.95	[0.91, 0.98]
Sex	0.256	1.59	[0.68, 3.62]			
GCS score	0.082	7.93	[3.63, 20.46]			
Systolic pressure admission	0.333	9.77	[1.76, 54.23]			
NIHSS score	0.452	4.88	[2.08, 18.32]			
The bleeding side of brain hemisphere	0.437	0.49	[0.28, 0.87]			
Hematoma volume	0.010^*^	5.69	[1.51, 10.69]	0.354	2.34	[0.91, 3.58]
Midline shift	0.378	1.01	[0.96, 1.06]			
Location	0.008^*^	6.91	[1.23, 10.21]	0.493	1.75	[0.69, 5.49]
The operative type	0.013^*^	5.34	[2.57, 18.58]	0.021^*^	2.69	[1.86, 4.06]
Operation time	0.476	0.23	[0.06, 0.39]			
Intraoperative blood loss	0.396	1.01	[0.96, 1.06]			
Hematoma clearance rate	0.358	0.96	[0.92, 1.03]			
Dose of mannitol (ml)	0.254	0.95	[0.86, 1.06]			
Complication	0.009^*^	0.16	[0.03, 0.72]	0.042^*^	0.36	[0.16, 1.68]
Postoperative duration of hospitalization	0.264	0.94	[0.83, 1.07]			

* *Statistically significant*.

## Discussion

With the current social improvement of living standards, more and more people are suffering from hypertension, and there are ~200 million patients with hypertension in China according to the latest epidemiological data (14). HICH is one of the most serious complications of hypertension because of its high morbidity and mortality rate, which accounts for 70–80% of a spontaneous cerebral hemorrhage. The mass effect of hematoma can cause primary brain injury in a short time and perihematoma edema is the main source of secondary cerebral damage (15). Therefore, surgical treatment has become an effective treatment for HICH patients because it can release the compression of the hematoma rapidly and reduce ICP at an early stage. A traditional craniotomy is not widely applied because of its disadvantages of severe trauma and postoperative complications. Currently, minimally invasive puncture drainage, small bone-window craniotomy, and neuroendoscopic hematoma evacuation are widely recognized by neurosurgeons. Because they have their advantages and limitations, experts and scholars have not reached a unified consensus about which of these surgical treatments is the most effective until now (16–18).

Nowadays, neuroendoscopic hematoma evacuation has been widely applied in the treatment of patients with HICH owing to its advantages of a clear operative field, short time, less bleeding, and injury (8, 9, 11, 19). However, there are also some limitations of the neuroendoscopic procedures, such as a narrow surgical window results in a low removal rate, sometimes cannot reach the designated position accurately and rapidly, and should be performed by a skilled surgeon. Hayashi et al. (11) reported that the surgeons should have experience doing the endoscopic procedure to achieve a good removal rate. Atsumi et al. (20) showed that the navigation system is beneficial for avoiding a burr hole exactly above the transverse and sigmoid sinus confirming the direction of hematoma extension in the neuroendoscopic surgery. For these reasons, we introduced the Remebot robot to improve clinical outcomes and prognosis of patients with HICH. The Remebot robot system has been successfully used in a variety of surgical methods that were designed and produced in China. We have examined the accuracy of the Remebot robot in several applications such as stereotactic brain biopsy and have shown it to be accurate (21). In addition, Wang et al. (14) showed that robot-assisted surgery using a Remebot is a safe and effective treatment method for hematoma removal and tube drainage in patients with HICH, and the target error is <1 mm. To the best of our knowledge, this is the first report to perform robot-assisted neuroendoscopic hematoma evacuation combined with ICP monitoring for patients with HICH. In this study, we found that the operation time of the procedure of the neuroendoscopic group was significantly longer than that of the robot-assisted neuroendoscopic combined ICP monitoring group (mean time 153.8 ± 16.8 vs. 132.8 ± 15.7 min, *P* < 0.001) and the intraoperative blood loss was significantly less in the robot-assisted neuroendoscopic combined ICP monitoring group than in the neuroendoscopic group (215.4 ± 28.3 vs. 190.1 ± 25.6 ml, *p* = 0.001). The Remebot robot navigation system has several benefits. First, because a supine lateral position and other positions can lead to disorientation, the robot system could help us to confirm whether the selection of the site of the burr hole was satisfactory. Second, it could help us better confirm the direction of hematoma extension and the depth to complete the hematoma extraction, which can reduce the number of punctures and trauma of most puncture wounds.

Moreover, postoperative management has important value for the recovery of patients with HICH. As is well-known, ICP monitoring has been widely used in the field of head trauma and neuro-critical care. MacLaughlin et al. (22) found that ICP monitoring was associated with a significant decrease in mortality rate through a retrospective analysis of 123 patients with severe traumatic brain injury. Many researchers also showed that ICP monitoring is crucial in the postoperative management of patients with HICH because it can improve postoperative treatment and prognosis by detecting the information of any abnormal increase of ICP in time (13). The application of mannitol drugs could reduce ICP of the patients with HICH after surgery and reduce the risk of rehemorrhage and brain edema. However, excessive use of mannitol drugs increases the risk of complications such as electrolyte disturbances, the imbalance of body fluid, and acute renal failure that can result in rapid clinical deterioration and increase the length of recovery. Without ICP monitoring, we made the postoperative treatment decisions that depends on clinical signs and imaging methods. In our study, we found that the postoperative duration of hospitalization of the neuroendoscopic group was significantly longer than the robot-assisted neuroendoscopic combined ICP-monitoring group (mean 13.8 ± 3.3 vs. 11.1 ± 2.8 days, *p* = 0.016), the dose of used mannitol was significantly less in the ICP monitoring group (615.2 ± 63.8 vs. 547.8 ± 65.3 ml, *p* < 0.001). In the meantime, there was no significant difference in mortality rate between the two groups, but there was a significant difference in mRS score at discharge, patients with less mRS score in the robot-assisted neuroendoscopic combined ICP-monitoring group than in the neuroendoscopic group (3.0 ± 1.0 vs. 3.8 ± 0.8, *p* = 0.011). The results showed that ICP monitoring could significantly reduce the postoperative duration of hospitalization and mannitol use because it can help the clinicians to initiate intervention timely before the irreversible cerebral damage. The complications rate was greater in the endoscopic group (25%) as compared with the robot-assisted neuroendoscopic combined ICP-monitoring group (9.5%) but without significant difference (*p* = 0.159). This could be explained by the limited number of patients.

Furthermore, patients undergoing robot-assisted neuroendoscopic combined ICP monitoring had better 6-month functional outcomes, and there was a significant difference between the groups (*P* = 0.004). Besides, multivariable analysis shows younger age, no complication, and robot-assisted neuroendoscopic combined ICP monitoring were predictors of 6-month favorable outcomes for the patients with HICH. The results are in accordance with those of previous literature. Che et al. (12) reported that a good 6-month functional recovery was associated with ICP monitoring. Ferrete-Araujo et al. (23) performed a prospective observational study of 186 patients with severe ICH, which showed that ICP monitoring and early operation were predictors of longer survival and better functional outcomes. For these reasons, we proposed that robot-assisted neuroendoscopic hematoma evacuation combined with ICP monitoring should be used for patients with HICH. Because it has the advantages of less operation time and intraoperative blood loss, less mannitol use and postoperative duration of hospitalization, and better functional recovery.

There are several limitations of our study. The primary limitations are its retrospective design and the number of patients enrolled was still not large enough, which limits the power of statistical tests. Moreover, the findings are limited by the presence of selection bias, as described previously, and the lack of multicenter participation. Therefore, a prospective, randomized controlled trial is needed to further evaluate the effect of robot-assisted neuroendoscopic hematoma evacuation combined with ICP monitoring for the treatment of HICH.

## Conclusion

Robot-assisted neuroendoscopic hematoma evacuation combined with ICP monitoring appears to be safer and more effective than neuroendoscopic hematoma evacuation in the treatment of HICH. Robot-assisted neuroendoscopic hematoma evacuation combined with ICP monitoring might improve the clinical effect and treatment outcomes of the patients with HICH.

## Data Availability Statement

The raw data supporting the conclusions of this article will be made available by the authors, without undue reservation.

## Ethics Statement

This study was approved by the Ethical Committee of Tongji Hospital, Tongji Medical College, Huazhong University of Science and Technology, China. The patients/participants provided their written informed consent to participate in this study. Written informed consent was obtained from the individual(s) for the publication of any potentially identifiable images or data included in this article.

## Author Contributions

KS contributed to the conception and design of this study. SW and HW contributed to data analysis and wrote the manuscript. WJ and TL contributed to the data analysis and to modify the article. FH and JW contributed to the data collection and data interpretation. All authors read and approved the final manuscript.

## Funding

This study was funded by the Research-type Clinician funding plan of Tongji Medical College, Huazhong University of Science and Technology (Grant No.5001540025).

## Conflict of Interest

The authors declare that the research was conducted in the absence of any commercial or financial relationships that could be construed as a potential conflict of interest.

## Publisher's Note

All claims expressed in this article are solely those of the authors and do not necessarily represent those of their affiliated organizations, or those of the publisher, the editors and the reviewers. Any product that may be evaluated in this article, or claim that may be made by its manufacturer, is not guaranteed or endorsed by the publisher.
